# Management of severe dengue hemorrhagic fever and bleeding complications in a primigravida patient: a case report

**DOI:** 10.1186/s13256-016-1129-7

**Published:** 2016-12-20

**Authors:** Hori Hariyanto, Corry Quando Yahya, Primartanto Wibowo, Oloan E. Tampubolon

**Affiliations:** 1 Department of Anesthesiology and Critical Care Medicine, 3rd floor, Siloam Hospitals Lippo Village, Jalan Siloam No. 6, Karawaci, 15811 Tangerang, Banten Indonesia; 2 Department of Anesthesiology, Universitas Pelita Harapan Faculty of Medicine, Jalan Boulevard Jendral Sudirman, Lippo Karawaci, Tangerang, 15811 Indonesia

**Keywords:** Dengue hemorrhagic fever, Bleeding, Pregnancy, Transfusions, Case report

## Abstract

**Background:**

The incidence of dengue hemorrhagic fever is increasing among the adult population living in endemic areas. The disease carries a 0.73% fatality rate for the general population, but what happens when the disease strikes a special subpopulation group, the obstetrics? Perhaps the important question specific to this special subpopulation revolves around the right time and mode of delivery under severe coagulopathy and plasma leakage in conditions of imminent delivery.

**Case presentation:**

A 24-year-old primigravid Sundanese woman presented to our intensive care unit due to acute pulmonary edema secondary to massive plasma leakage caused by severe dengue. She tested positive for both immunoglobulin G and immunoglobulin M dengue serology indicating she had secondary dengue infection, which placed her at risk for an exaggerated cytokine response as was evident clinically. She had to undergo an emergency cesarean section which was later complicated by rebleeding and hemodynamic instability due to an atypical defervescence period. She was successfully managed by multiple blood transfusions and was discharged from our intensive care unit on day 8 without any negative sequel.

**Conclusions:**

Fever, thrombocytopenia, and hemoconcentration are the classical symptoms of dengue hemorrhagic fever observed in adult, pediatric, and obstetric populations. However, a clinician must be particularly watchful in treating a pregnant dengue-infected patient as physiologic hematology changes provide greater volume compensation and the advent of shock marks significant volume loss. In conclusion, an important principle in the management of dengue hemorrhagic fever in pregnancy is to prioritize maternal well-being prior to addressing fetal issues.

## Background

Dengue is a common tropical infectious disease with a rising incidence among the Indonesian population. In 2013, its annual incidence was reported to be 35 to 40/100,000 with a mean age of above 15-years old and a case fatality rate of 0.73% [1]. The classical form of dengue infection is manifested as high fever, violent headaches, thrombocytopenia, and hemoconcentration. Such presentations are similar to those within the obstetric population; however, bleeding tendency is increased especially for both the mother and the neonate due to hemostatic defects which might lead to uncontrolled bleeding [2].

The management of dengue hemorrhagic fever during pregnancy warrants careful monitoring as physiologic hemodilution may mask hemoconcentration leading to late diagnosis and late management of the severely volume-depleted patient. Plasma leakage accumulates along the tissue interstitial space and causes inadequate tissue oxygenation. Prolonged disturbance may develop into multiorgan failure and rapid fetal demise especially in the obstetric population where oxygen consumption is twice as high as the healthy adult [3].

Fluid therapy and identification of the critical phase are the most important aspects of management, but what does clinical evidence say about dengue infection in the obstetric population? Diagnosis of dengue infection during pregnancy surely affects management options and decisions as the mode and time of delivery are of utmost importance. In this case report, we will discuss the pathophysiology and management of severe dengue hemorrhagic fever and bleeding complications in an intensive care unit (ICU).

## Case presentation

A 24-year-old Sundanese primigravid woman was referred from a peripheral hospital at 38 weeks of gestation due to her deteriorating condition. She presented initially for 5 days of high grade fever, retro-orbital pain, and a blood examination which revealed thrombocytopenia, elevated liver enzymes, and a positive immunoglobulin M (IgM) and immunoglobulin G (IgG) dengue serology. She was diagnosed as having dengue fever in pregnancy and treated with fluid administered intravenously and antipyretics. However, her condition started to worsen on day five of hospitalization with repeated bouts of vomiting and she became lethargic. Her weight was 45 kg and her antenatal history did not reveal hypertension, pre-eclampsia, coagulation abnormalities, or epilepsy.

**Fig. 1:**
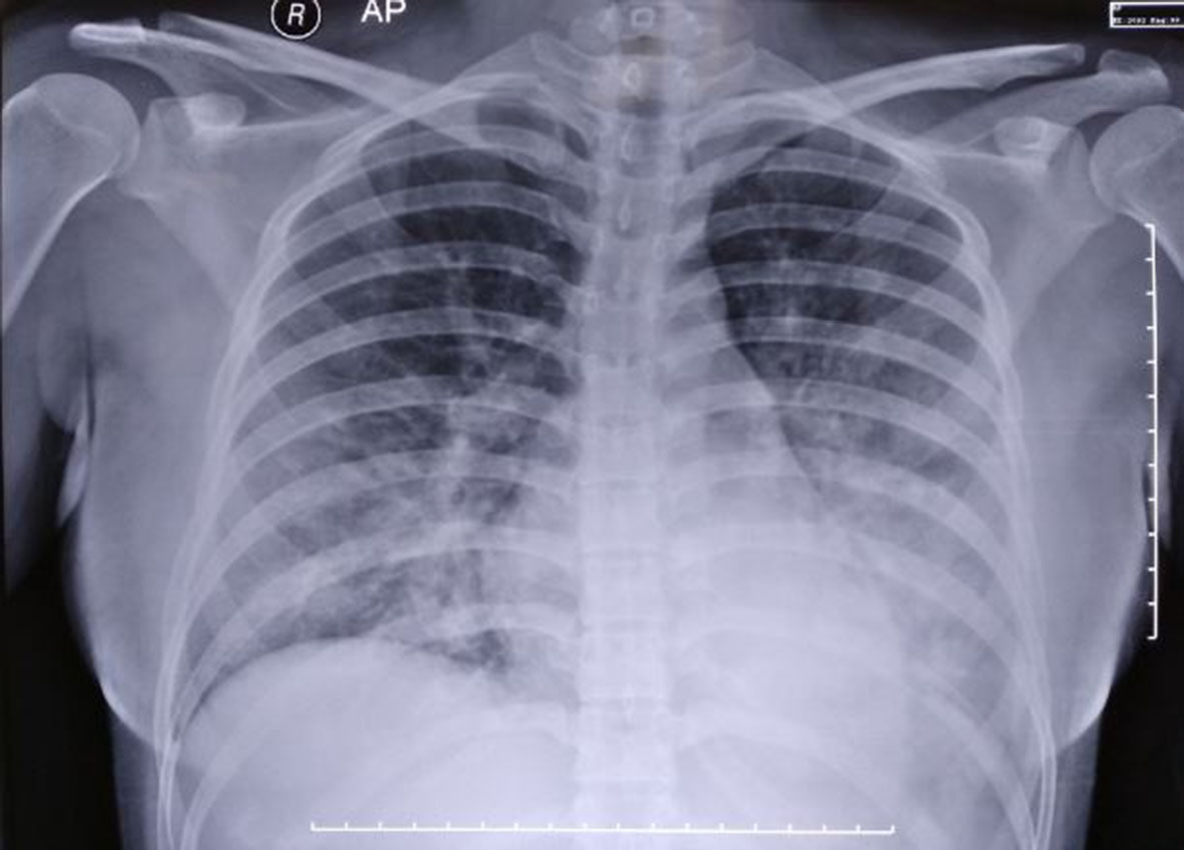
Chest X-ray upon initial admission

During her transport, she received 10 liters of oxygen by non-rebreathing mask and had experienced two episodes of generalized tonic–clonic seizure, each lasting less than 1 minute which terminated with 10 mg diazepam administered intravenously. On admission to our ICU, she was responsive only to pain with a blood pressure of 92/76 mmHg, heart rate 124/minute, respiratory rate 30/minute, body temperature 36.6 °C, and oxygen saturation of 95%. A physical examination revealed diffuse rales on both lungs with cold and clammy extremities. Her chest X-ray revealed marked bronchovascular marking on her left and right basal lung regions (Fig. 1). Arterial blood gas analysis revealed an acute metabolic alkalosis: pH 7.510, partial pressure of oxygen in arterial blood (PaO_2_) 166 mmHg, partial pressure of carbon dioxide in arterial blood (PaCO_2_) 41.3 mmHg, bicarbonate (HCO_3_^-^) 33.0 mmol/L, and base excess (BE) 9.3 mmol/L. An initial diagnosis of dengue encephalitis, and dengue shock syndrome with acute pulmonary edema was made.

She was immediately intubated and placed on mechanical ventilation using adaptive support ventilation mode with the following settings: minute ventilation of 4.5 L, positive end-expiratory pressure (PEEP) 5 cmH_2_O, and fraction of inspired oxygen (FiO_2_) of 50% and received continuous sedation under morphine and midazolam infusion. During anesthesia induction, her blood pressure dropped to 60/40 mmHg and a fluid bolus of 300 mL normal saline was given to which she responded. Arterial blood gas analyses taken 2 hours post-intubation with a FiO_2_ 0.7 were as follows: pH 7.475, PaO_2_ 159.1 mmHg, PaCO_2_ 32.1 mmHg, HCO_3_^-^ 24.2 mmol/L and BE 1.1 mmol/L. Another important event included traumatic gum bleeding from biting during her seizure episode which took 1 hour to attain hemostasis, and insertion of a nasogastric tube which yielded 100 mL of dark brown fluid.

On day 1, hematologic results revealed hemoglobin (Hgb) of 11.7 g/dL (normal range, N, 11.70 to 15.50 g/dL), hematocrit (Hct) of 36.80% (N, 35.00 to 47.00), and white cell count (WBC) of 10,430/mm^3^ (N, 3600 to 11,000/ mm^3^) with 53% neutrophil predominance and 35% lymphocytes. Her platelet (Plt) count was 25,000/uL (N, 150,000 to 440,000), prothrombin time (PT) 10.40 seconds (N, 9.4 to 11.3) with an international normalized ratio (INR) of 1.00, activated partial thromboplastin time (aPTT) of 48.70 (N, 31 to 45 seconds), and a slightly elevated D-dimer of 1.91 ng/mL (N, 0.00 to 0.30). Her bilirubin levels were normal, she had an alanine aminotransferase (ALT) level of 116 U/L (N, 0 to 55), aspartate transaminase (AST) of 359 U/L (N, 5 to 34), urea of 49.0 mg/dL (N, <50), creatinine of 0.85 mg/dL (N, 0.5 to 1.1), lactic acid of 4.7 mmol/L (N, <0.6 to 2.2) and procalcitonin of 0.25 ng/mL (N, <0.15). Her urine was tinted red and complete analysis revealed the presence of slight proteinuria (100 mg/dL) and occult blood (200 cells/uL). Cardiotocography (CTG) monitoring revealed a fetal heart rate of 177 beats per minute (bpm) with no uterine contractions.

On day 2, a routine CTG monitoring revealed fetal distress which prompted an emergency cesarean section. She received 500 mL fresh frozen plasma (FFP) before being rushed for cesarean section under general anesthesia. Intraoperative bleeding was 500 mL, an intra-abdominal drain was placed and she received 460 mL of packed red cell (PRC), intraoperatively. A 2.1 kg baby girl was delivered with an appearance, pulse, grimace, activity and respiration (APGAR) score of 4/7. The baby was intubated and transferred to our neonatal ICU (NICU) due to hypoventilation. Our patient was transferred back to ICU and fundal height was noted at the level of umbilicus with good contractions.

**Fig. 2:**
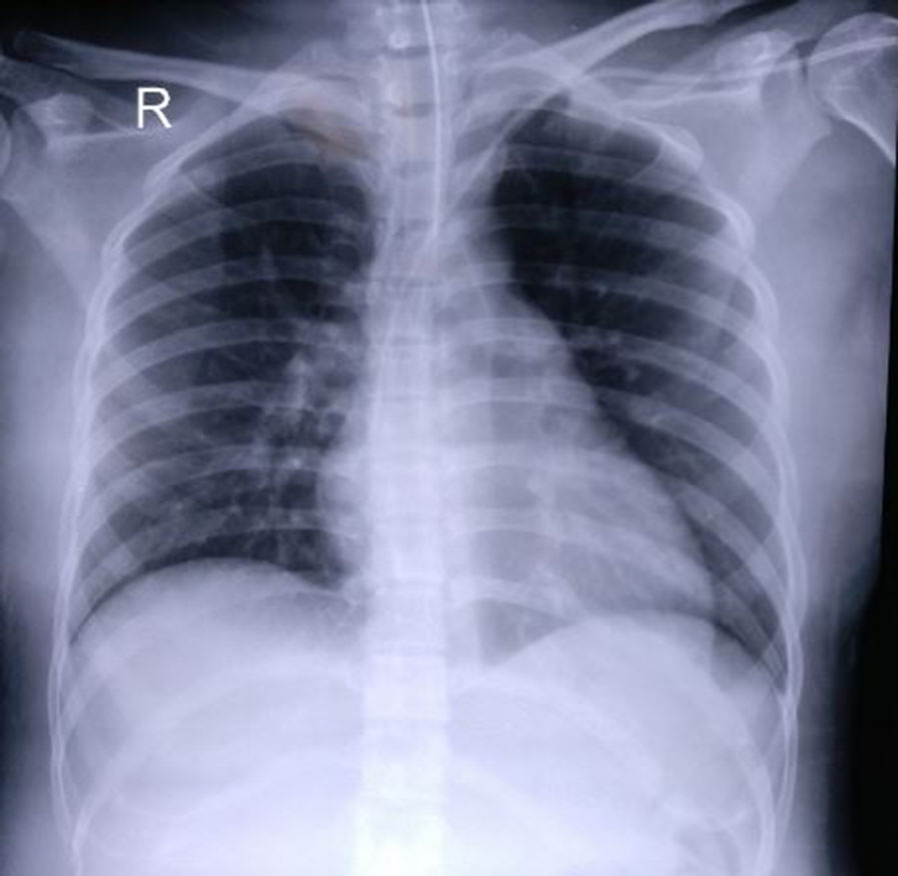
Chest X-ray on day 3 of hospitalization

On day 3, blood clots were seen oozing from her vagina and a vaginal exploration evacuated 200 mL of blood. The intra-abdominal drain collected 50 mL/24 hours of hemoserous fluid and hematologic results revealed Hgb 5.9 g/dL, Hct 18.20%, and Plt 141,000/uL. Upon this substantial drop, 680 mL of PRC, 210 mL of FFP, and 2 units of thrombocyte concentrate apheresis (TC_A_) were given. Her vital parameters were stable and she remained sedated. A follow-up chest radiography revealed improved clearance of vascular markings on both lung fields confirmed by vesicular lung sounds and a normal acid-base balance with a PaO_2_/FiO_2_ of 600 (Fig. 2).

On day 4, a follow up hematologic examination revealed Hgb 6.96 g/dL, Hct 20.16%, WBC 10,660/mm^3^, Plt 98,630/uL, albumin 2.63 g/dL, fibrinogen 140 mg/dL (N, 300 to 600), PT 10.90 seconds, and a prolonged aPTT of 56.90 seconds. The intra-abdominal drain collected 450 mL/24 hours of hemoserous fluid and her lower abdomen was noted to be slightly distended. Thereafter, another 230 mL of PRC, 240 mL of FFP, and 2 units of thrombocyte concentrate (TC) was transfused.

On day 5, 3 days post-cesarean section, our patient became tachycardic with a blood pressure of 80/60 mmHg, which prompted the use of norepinephrine at 0.08 μg/kg/minute. Uterine contractions were adequate with normal colored lochia, but her lower abdomen appeared distended as before. The intra-abdominal drain collected 360 mL/24 hours of hemoserous fluid and laboratory results were as follow: Hgb 5.90 g/dL, Hct 17.80%, WBC14,020/mm^3^, Plt 107,000/uL, and albumin of 2.26 g/dL. She was given 20% albumin infusion, 230 mL of PRC, and 220 mL of FFP.

On day 6, she remained tachycardic and her mentation did not improve despite stopping sedation. At this time, her lower abdomen appeared more distended and abdominal guarding was noted upon palpation. The intra-abdominal drain had collected 850 mL/24 hours of hemoserous fluid. An urgent blood workup revealed a drop in Hgb to 4.20 g/dL, Hct 12.50%, WBC 10,160/mm^3^, Plt 84,000/uL, PT 10.40 seconds, aPTT 49.50 seconds, and a spiked D-dimer level (20.25 ug/mL). An emergent ultrasound revealed free fluid on her lower abdominal region which required her to be rushed for an exploratory laparotomy. The procedure evacuated 2000 mL of blood intra-abdominally, and she received 640 mL of PRC and 500 mL FFP, intraoperatively.

On day 7, her hematologic examination revealed Hgb 9.60 g/dL, Hct 28.50%, WBC 8,150/mm^3^, Plt 67,000/uL, normalized coagulation profile, and a lowered D-dimer level (13.98 ug/mL). One unit of TC was transfused and norepinephrine infusion was tapered down with strict vital sign monitoring. Sedation was stopped and her mentation greatly improved. A follow-up chest radiograph revealed clear lung fields and she was placed on a spontaneous breathing trial which was successful and she was later extubated.

On day 8, an additional 230 mL PRC was given and her hematologic examination revealed Hgb 11.50 g/dL, Hct 34.40%, and Plt 83,000/uL. Aside from transfusions and vasopressors, she received meropenem 1 gram every 8 hours, early enteral nutrition of 1000 kcal/24 hours, gastric acid prophylaxis agent, gastric motility agents, and anticonvulsants. She was transferred to our general ward on day 8 and discharged from our hospital on day 11, uneventfully. Her neonate was extubated on day 2 of her NICU stay, after complete recovery from sedative drugs *in utero* and discharged home at day 5 without any negative sequel (Table 1).

**Table 1:** Daily hematology trend and therapy

		Day 1	Day 2	Day 3	Day 4	Day 5	Day 6	Day 7	Day 8
Therapy given prior to blood test results	Packed red cell (mL)	None	460	None	None	None	None	None	230
	Thrombocyte concentrate apheresis (Unit)	None	None	None	None	None	None	None	None
	Fresh frozen plasma (mL)	None	500	None	None	None	None	None	None
Hemogram	Hemoglobin (g/dL)	11.7	13.3	5.9	6.96	5.9	4.2	9.6	11.5
	Hematocrit (%)	36.8	41.9	18.2	20.16	17.8	12.5	28.5	34.4
	White blood cell (/mm^3^)	10,430	10,690	11,390	10,660	14,020	10,160	8150	
	Platelets (/μL)	25,000	31,000	141,000	98,030	107,000	84,000	67,000	83,000
Coagulation studies	Prothrombin time (seconds)	10.4	10.9	12.1	10.9	10.4	10.4	9.9	10.2
	Activated partial thromboplastin time (seconds)	48.7	46.1	51.7	56.9	55.2	49.5	45.6	41.5
	D-dimer (μg/mL)	1.91	2.71	1.49	5.36	18.96	20.25	13.98	11.2
	Fibrinogen (mg/dL)	N/A	N/A	N/A	140	N/A	N/A	N/A	N/A
Therapy given after blood results	Packed red cell (mL)	None	None	680	230	230	640	None	None
	Thrombocyte concentrate apheresis (Unit)	None	None	2	2	None	None	1	None
	Fresh frozen plasma (mL)	None	None	210	240	220	500	None	None
Amount of blood collected + abdominal drain (mL/24 hours)			500 (cesarean section)	50	450	360	850 + 2000 evacuated intra-abdomen	Minimal	Minimal

*N/A* not available

## Discussion

Dengue infection presents with a febrile period of 2 to 7 days followed by 3 to 4 days of defervescence phase marked by massive plasma leakage which may progress to shock [4]. Recall that during pregnancy, the body experiences physiologic changes with respect to the cardiovascular, respiratory, and hematologic systems [5]. At the end of the third trimester, plasma volume increases by approximately 40% resulting in dilutional anemia which masks the ‘hemoconcentration’ commonly observed during the defervescence phase of dengue hemorrhagic fever. The finding is confirmed as our patient was at her tenth day of illness. She was at the ‘critical’ phase of dengue infection with a normal Hct; nevertheless, she was in a state of shock with altered mental status and reduced perfusion.

Severe manifestations of dengue infection are reported to be a combination of factors from the host, viral virulence, and those presenting with secondary exposures [6]. The dengue virus replicates intracellularly and carries tropism to endothelial cells, pulmonary cells, and gastrointestinal cells which triggers an antigen-antibody complex causing immune-mediated cell destruction and the production of cytokines and antibodies [7]. Our patient tested positive on IgG and IgM serology test, thus categorizing her with secondary dengue infection, which increases the risk for an exaggerated cytokine cascade response; this response was made clinically evident by severe thrombocytopenia (<50,000), elevated liver enzymes, mucosal bleeding, and massive plasma leakage which contributed to the development of acute pulmonary edema [8].

On arrival to our ICU, our patient was only responsive to pain stimulation with significant tachypnea and labored breathing. In pregnancy, the normal arterial pH is 7.45 with a PaCO_2_ of 30 mmHg due to increased minute ventilation. Our patient revealed alkalosis with a rising paCO_2_ level of 41.3 mmHg indicating that she was developing fatigue marked by CO_2_ retention and impaired ventilation [5]. Marked vascular markings on radiologic examination and crackles upon lung auscultation signify a potential disruption in the process of oxygenation. More importantly, the obstetric patient requires a greater amount of oxygen to meet her basal metabolic rate and every pregnant patient is considered at risk for aspiration due to reduced gastric emptying time [5]. For these reasons, she was intubated and supported by mechanical ventilation.

Perhaps the most important question to ask revolves around the mode and timing of delivery of a severely thrombocytopenic patient. Should delivery be performed even before the onset of fetal distress and what are the recommended guidelines on thrombocytopenia in pregnancy? Two units of FFP were transfused as recommended by World Health Organization transfusion guidelines prior to elective surgical procedures [9, 10]. We believe cesarean section performed on our patient due to fetal distress was the appropriate decision. On her arrival to our institution, optimization of maternal status was aggressively pursued in order to provide optimal oxygen delivery to the fetus. Unfortunately, the fetus’s condition deteriorated which may have been a result of prior maternal hypoxemia and alkalotic condition disrupting oxygen release to tissue. General anesthesia was the preferred mode of delivery as neuraxial anesthesia was impossible owing to our patient’s respiratory distress and thrombocytopenia [11]. After the operation, she was given 2 units of PRC and an intra-abdominal drain was inserted in anticipation of bleeding diathesis.

Over the next few days, insidious bleeding occurred as her Hgb substantially plummeted from 13.3 g/dL to 4.2 g/dL within 4 days. Even though thrombocytopenia is universally observed in dengue hemorrhagic fever, it is a poor indicator of bleeding manifestation. It is rather the low fibrinogen levels and prolonged aPTT which are the main culprits for coagulopathy [12]. Normal pregnancy is a state of hypercoagulopathy due to a physiologic increase in coagulation factors [13]. By contrast, our patient had lowered fibrinogen levels and prolonged aPTT. This is caused by increased vascular permeability causing leakage of fibrinogen into the interstitial spaces while pronounced cytokine response causes damage to glycocalyx along the endothelial linings and liberates heparin sulfate into the circulation, thereby disrupting the intrinsic coagulation pathway [14]. Such disturbance combined with a recent operative procedure triggered the ongoing hemorrhage in our patient.

The critical phase of dengue hemorrhagic fever lasts 2 to 4 days, but our patient continued to face hemostatic defects and volume depletion up to her sixth day. During her secondary infection of dengue, the binding of the new virus to cross-reactive antibodies from her previous infection resulted in the uptake of virus into mononuclear phagocytes. This enables the virus to amplify viral replication resulting in a higher viral antigen load and an exaggerated form of coagulopathies and vascular permeability [7, 15]. The plasma leakage is caused by the functional disruption of adherens junction, a network of adhesion proteins in the intracellular cytoskeleton that retract and create gaps between cells promoting leakage of plasma and coagulation proteins, which is transient and resolves itself as the cytokine response wears off [6].

Throughout the course of her ICU stay, our patient received a total of 11 units of PRCs, 7 units of FFP, 2 units of TC_A_, and 3 units of TC. Apart from blood product transfusions, treatment of severe dengue hemorrhagic fever includes judicious fluid therapy during the ‘critical’ phase to avoid leakage of fluids into the fragile pulmonary capillaries. Hence, we used 20 ml/hour of normal saline infusion while striving for zero fluid balance and maintaining a diuretic goal of 1 mL/kg/hour. Stable vital signs, clearance of vascular markings on the chest X-ray, and a normalized PaO_2_/FiO_2_ ratio indicated appropriate fluid titration in our patient.

## Conclusions

Current evidence has reported similarities in symptoms and laboratory findings in the obstetric population infected by dengue hemorrhagic fever. However, clinicians must be aware that secondary dengue infections may manifest an atypical period of defervescence phase marked by severe prolonged coagulopathies and plasma leakage. Hence, the management of dengue-infected obstetric patients is aimed at conservative fluid therapy and transfusion of blood product when signs of bleeding occur. An important principle to remember is to prioritize maternal well-being prior to addressing fetal issues. Next, a delivery plan via the fastest route and timely administration of blood products prior to delivery is essential in order to deliver the most beneficial outcome for both the mother and fetus.
